# Focusing on modifiable early protective factors to prevent negative neurodevelopmental and psychiatric outcomes in at-risk infants

**DOI:** 10.3389/fpsyt.2023.1302474

**Published:** 2023-12-08

**Authors:** Michele Poletti, Antonio Preti, Andrea Raballo

**Affiliations:** ^1^Child and Adolescent Neuropsychiatry Service, Department of Mental Health and Pathological Addiction, Azienda USL-IRCCS di Reggio Emilia, Reggio Emilia, Italy; ^2^Department of Neuroscience, University of Turin, Turin, Italy; ^3^Chair of Psychiatry, Faculty of Biomedical Sciences, University of Southern Switzerland, Lugano, Switzerland; ^4^Cantonal Sociopsychiatric Organisation, Mendrisio, Switzerland

**Keywords:** genetic risk, familial, mental illness, protective factor, parenting

## Introduction

In the scientific debate, there is increasing awareness of the relevance of the first years of life as a basis for health and disease, as stated by the Developmental Origins of Health and Disease (DOHAD) hypothesis (1). Early at-risk conditions are determined by multiple potential genetic and environmental factors, as clearly exemplified by preterm birth. Preterm children are at increased risk of abnormal physical health and neurobehavioral development (2, 3), including increased long-term risk of psychiatric illness (4), with a gradient of risk related to the grade of prematurity (i.e., gestational age at birth). At the same time, preterm birth and obstetric complications are over-represented in the developmental anamnesis of adult patients with severe mental illnesses (5–7), in particular, schizophrenia (5). Integrating prospective (from the point of view of preterm birth) and retrospective (from the point of view of the psychiatric diagnosis) perspectives, preterm birth is indeed considered a potential early trigger for altered neurodevelopment, leading to the structuration of a schizophrenic vulnerability in adolescence and young adulthood (8, 9).

## G × E

Each early risk factor, whether of genetic, epigenetic, familial, or environmental origin, is likely not deterministically sufficient to cause full-blown clinical psychiatric outcomes during development. Instead, its effects are plausibly amplified through interactions with other early risk factors and later supervening factors, ultimately surpassing adaptive biopsychosocial thresholds. This is the overall picture that emerges from twin (10) and adoption studies on schizophrenia (11). For example, the Finnish Adoptive Developmental Study (12) compared a sample of offspring (*n* = 185) of schizophrenic mothers who were adopted within their 4th year of life to matched controls in which the adopted children had no biological parents diagnosed with schizophrenia. These findings indicated an interaction between genetic risk factors and protective environmental factors, with a dimensional distribution of the risk of developing schizophrenia. Thus, regardless of the genetic risk (presence or absence of schizophrenia in the biological mother), children raised in adoptive families without severe mental health problems showed minimal levels of psychopathology over time. On the contrary, the level of psychopathology increased if an adoptive parent suffered from mental disorders and even more so if both parents were ill. This pattern is intriguingly in agreement with experimental findings derived from animal models showing that adoption may reverse the effects of early perinatal stress (13).

## Families with mental illness as hyper-aggregators of risk factors

From this perspective, families affected by psychiatric illness represent a peculiar ecological niche in which the intergenerational transmission of severe mental disorders is boosted by the hyper-aggregation of genetic plus early and late environmental risk factors (14, 15). These include the additive maternal and paternal genetic load in the case of non-random (assortative) mating between individuals with severe mental disorders (16) and early environmental risk factors, such as *in utero* exposure to pharmacological agents (17) and obstetric complications like preterm birth, which, incidentally, are both more prevalent in pregnancies of mothers with mental illnesses (18). Moreover, in the early years, enduring chronic familial non-protective conditions may amplify the effects of early genetic and environmental risk factors. Within families, risk factors, indeed, include heightened expressed emotions (19), prolonged familial distress (20, 21), and inadequate parenting (22), while factors outside the family include perceived psychosocial inadequacy in comparison to peers and social stigma. Among these familial risk factors that may contribute to the perpetuation of intergenerational liability to psychopathology, parenting is a crucial one. Indeed, the quality of parenting is heavily conditioned by the severity and chronicity of mothers' mental illness (23), yet it is also potentially modifiable through psychosocial interventions.

Overall, because many pathogenic environmental factors originate from the familial environment, which may be partially determined by their genetics, severe mental illnesses can be considered familial (24). In this case, familiality does not overlap with Mendelian genetics and possibly relies on epigenetic modifications dependent on early-life social environment and mental health (24).

## Parenting as a modifiable factor

Inadequate parenting related to serious mental illness is implicated in increased rates of insecure or disorganized attachment patterns, which, combined with an underlying neurobiological vulnerability, may further exacerbate the lifetime risk of developing schizophrenia (25). However, despite the increasing amount of evidence on the potential role of parental mental illness in parenting, there is a relative paucity of high-quality studies addressing interventions to support parenting in psychiatric parents. A recent meta-analysis of randomized controlled trials quantified the effects of preventive interventions for this at-risk population, reporting small though significant Effect Sizes (ES) for programs enhancing the mother-infant interaction (ES = 0.26) as well as mothers' (ES = 0.33) and childrens' (ES = 0.31) behavior, which proved to be stable over a 12-month follow-up (26).

Therefore, parenting is a modifiable variable, and its early protective factor has been demonstrated by a recent prospective, observational cohort study (27) providing a significant step forward in the empirical investigation of the role of the quality of mother-child interaction in moderating early brain development and longitudinal endophenotypic neurocognitive outcomes in preterm children. The authors recruited 226 infants born preterm (i.e., below 32 weeks of gestational age) from a neonatal intensive care unit to examine whether maternal sensitivity at 3 years buffers the relationship between cortical gray matter dysmaturation early in life and at term-equivalent age and cognitive and language abilities at 3 years (after controlling for neonatal illness, brain injury, and maternal education). They found that small for gestational age preterm children, as well as the presence of postnatal infections or brain injuries, was associated with lower neurodevelopmental scores; moreover, better supportive maternal behavior sensitivity was associated with better cognitive and language outcomes in children with less mature cortical gray matter at term-equivalent age. Therefore, they concluded that supportive maternal behavior following early brain dysmaturation may provide an opportunity to promote optimal neurodevelopment in preterm children. A similar protective effect provided by supportive maternal behavior could be hypothesized and tested for children of schizophrenic mothers, who, at neuroimaging, may present prenatal and neonatal signs of brain dysmaturation in comparison with age-matched children of healthy mothers (28).

## Conclusions

In sum, children of parents with severe mental illness represent a paradigmatic example of an *ab origine* at-risk category that combines putative genetic liability with early, enduring, and potentially increasing over-exposure to multiple environmental risk factors. This risk is confirmed by a meta-analytical increased risk of mental illness in the offspring of parents with mental illness (29, 30).

The intertwining of risk factors exemplifies how, at least in this field, familial risk factors are not simply genetic in nature but also involve the early relational milieu. Since the pathogenic early postnatal factors stem from the familial environment, mental illness can be characterized as having familial roots. Consequently, there may be a familial component in the pathological phenotype triggered by early-life stress. In this specific scenario, familiality is distinct from Mendelian genetics and likely hinges on epigenetic modifications, strongly indicating that “genetic” and “familial” are not interchangeable terms (24).

Among environmental factors, parenting is crucial and may be modifiable and have a protective effect. Indeed, in the long term, good parenting has the potential to mitigate lifetime psychiatric risk, as demonstrated by adoption studies (11, 12), whereas, in the short term, it seems to potentially mitigate the neurocognitive effects of altered neurodevelopment due to early environmental adversities, such as preterm birth (27). Therefore, maternal sensitivity has concrete effects not only for the positive mother–child bond and for a secure attachment (which exerts a broad protective role in the cognitive and socioemotional development of infants) but also for the development of cognitive functions despite early brain dysmaturation due to preterm birth (31). This strongly supports the rationale for developing primary preventive programs to support parenting, especially supportive maternal behavior, in at-risk mother–child dyads (for example, for maternal mental illness, child vulnerability, or both) to support a cascade of beneficial effects, from the neural to the cognitive and psychological levels.

## Author contributions

MP: Conceptualization, Writing – original draft, Writing – review & editing. AP: Conceptualization, Supervision, Writing – review & editing. AR: Conceptualization, Supervision, Writing – review & editing.
